# Data-Driven Hazardous Gas Dispersion Modeling Using the Integration of Particle Filtering and Error Propagation Detection

**DOI:** 10.3390/ijerph15081640

**Published:** 2018-08-02

**Authors:** Zhengqiu Zhu, Sihang Qiu, Bin Chen, Rongxiao Wang, Xiaogang Qiu

**Affiliations:** 1 College of System Engineering, National University of Defense Technology, Changsha 410073, China; admin@steven-zhu.me (Z.Z.); nudtcb9372@gmail.com (B.C.); wangrx-nudt@foxmail.com (R.W.); michael.qiu@139.com (X.Q.); 2 Faculty of Electrical Engineering, Web Information Systems, Mathematics and Computer Sciences, Delft University of Technology (TU Delft), 2628 XE Delft, The Netherlands

**Keywords:** atmospheric dispersion, data-driven modeling, error propagation, particle filter, Gaussian dispersion model

## Abstract

The accurate prediction of hazardous gas dispersion process is essential to air quality monitoring and the emergency management of contaminant gas leakage incidents in a chemical cluster. Conventional Gaussian-based dispersion models can seldom give accurate predictions due to inaccurate input parameters and the computational errors. In order to improve the prediction accuracy of a dispersion model, a data-driven air dispersion modeling method based on data assimilation is proposed by applying particle filter to Gaussian-based dispersion model. The core of the method is continually updating dispersion coefficients by assimilating observed data into the model during the calculation process. Another contribution of this paper is that error propagation detection rules are proposed to evaluate their effects since the measured and computational errors are inevitable. So environmental protection authorities can be informed to what extent the model output is of high confidence. To test the feasibility of our method, a numerical experiment utilizing the SF_6_ concentration data sampled from an Indianapolis field study is conducted. Results of accuracy analysis and error inspection imply that Gaussian dispersion models based on particle filtering and error propagation detection have better performance than traditional dispersion models in practice though sacrificing some computational efficiency.

## 1. Introduction

Air contaminant emissions and contaminant gas leakage incidents in a chemical cluster pose a potential threat to public health and surrounding environment. Therefore, modeling atmospheric dispersion is a popular issue these years since it plays an important role in evaluating the impact of hazardous gas leak accidents [1,2]. Traditional methods (e.g., Gaussian-based dispersion models and Lagrangian dispersion models) usually use static model, wherein model parameters are pre-determined and invariant. However, due to the dynamic and stochastic nature of atmospheric dispersion, it is impractical to measure these model parameters precisely, especially the meteorological data (e.g., the wind field) [3]. Further, the computational error of static model may be accumulated with time during the calculation process. To address this problem, a data-driven modeling method is proposed in this paper for updating model parameters to generate the simulation results being as close as possible to the real data. However, some errors cannot be avoided, such as measurement errors and floating-point errors. Since these errors are inevitable, people have to know whether the predicted result is computed in an acceptable scope. Error propagation detection is a quite useful approach for error analysis during the computation. Consequently, it is necessary to develop an atmospheric dispersion modeling method that supports both data-driven modeling and error propagation detection.

Data-driven modeling methods based on data assimilation (DA) provide an approach of dynamically estimating model parameters and effectively improving the accuracy of model predictions. This approach assimilates the observations into the model to produce a time sequence of estimates of system states [4]. With the model parameters adjusted, the accuracy of the model prediction is consequently improved. Therefore, data-driven modeling methods based on DA have been widely used in various fields, especially in numerical weather forecasting and meteorological pre-processing. Some studies implemented data-driven modeling into short-range atmospheric dispersion [5,6]. They used Gaussian diffusion model and optimization method for minimizing the cost function to support parameter real time updating. A previous study used a particle filter and the European Tracer Experiment (ETEX) dataset to assimilate observations into an atmospheric transport model [7]. Data-driven modeling can also be used in atmospheric dispersion model to assess the impact of nuclear accidents [8]. Furthermore, Kalman filter and its extended methods are also extensively used in data-driven modeling due to its extensive framework [9,10]. A modified ensemble Kalman filter for nuclear accident prediction was proposed [11,12]. Reddy et al. utilized particle filter to improve diffusion model based on Gaussian multi-puff equation [13]. Among these methods, particle filter is one of the most suitable approaches for highly nonlinear and non-Gaussian models [14]. Using a series of weighted random sampling particles to approximate the posterior probability density function of the system state, particle filter is able to estimate arbitrary probability densities with few assumption constraints. Therefore, particle filter is applied as the data assimilation method in the air contaminant dispersion in this paper.

Error propagation is a troubling and common problem in numerical computation. The problem in atmospheric dispersion simulation is no exception. Many methods were proposed in previous studies to find how the computational error propagates [15,16,17]. Thus, researchers can then modify the model to reduce computational error according to error propagation detection results. In 1980, Ginsberg proposed a method to monitor floating-point error propagation in scientific computation [18]. Some researchers analyzed static program to find the floating-point accuracy problems [19,20,21,22,23]. Bao et al. developed an on-the-fly floating-point error propagation monitoring method to find the potential floating-point unstable errors [24]. A point system based on significance arithmetic was described in Sofroniou’s research [17]. Previous studies only gave analysis results marked as Boolean tag and just analyzed several common operations and statements. However, atmospheric dispersion modeling needs some advanced operations such as exponential and square root. Therefore, the error propagation detection applied in this paper is developed to support more operations and give multi-level results instead of Boolean tags.

The rest of this paper is organized as follows: Section 2 briefly introduces the data-driven modeling based on particle filter, Gaussian-PF model, error propagation detection and relative error exponent. In Section 3, a field study was utilized to illustrate the feasibility of our proposed method. Results and discussion are concluded in Section 4. Section 5 summarizes the conclusions.

## 2. Methods

### 2.1. Data-Driven Modeling Based on Particle Filter

Previously, Kalman Filter (KF) based methods work successfully in linear systems. However, when KF and its variations are used in a non-linear system, the system must be linearized. For most practical systems, the work of linearization is hard to complete. Fortunately, Particle Filter (PF), a suitable filtering method for non-linear system, can be extensively applied in data-driven modeling. PF uses a set of particles to update the system parameters via a Monte Carlo method [25]. Therefore, it can be used in complex systems that are difficult to linearize. Generally, the expression of a non-linear system is as shown as Equation (1):

(1) $$\left\{ \begin{array}{l} X_{k+1}=F(X_{k})+n_{k}\\ Z_{k+1}=H(X_{k})+v_{k} \end{array}\right.,$$

where $X_{k}$ represents the state condition at time step k, and $Z_{k}$ represents the observation at time step k, F and H are state transition function and observation function respectively. $n_{k}$ and $v_{k}$ are noises following Gaussian distribution, being added in state condition and observation respectively. When PF is applied in an atmospheric dispersion process, state condition $X_{k}$ means the combination of system parameters that can represent the current state of dispersion process. One of the common practices is dividing the area of dispersion into numerous grids and choosing the concentrations by grid as the state condition. This choice directly describes the atmospheric dispersion. However, the vast region of the chemical cluster means a high dimension of the state parameters, which results in a high computation cost. Observation $Z_{k}$ means the concentration dataset measured from monitoring stations. At each step of particle filtering, an expected state of dispersion process is estimated for forecasting the concentration distribution in next step. Furthermore, the state transition function H should be fast due to the real-time requirement of the dynamic data-driven modeling, so Gaussian dispersion model is a quite appropriate option.

### 2.2. Gaussian-PF Model

In Gaussian dispersion model, most parameters (e.g., wind speed, wind direction, source term and etc.) can be obtained by monitoring data (i.e., observations or measurements in this paper). However, the diffusion coefficients are very difficult to be directly measured, which are affected by environmental conditions (e.g., diffusion terrain, atmospheric stability, and sunlight). Generally, diffusion coefficients can be expressed by the function of downwind distance and atmospheric stability class. Therefore, the diffusion coefficients are chosen as the system states to realize data-driven modeling.

Gaussian plume model is utilized for atmospheric dispersion modeling with constant release rate and static wind field. To obtain the expression, the following assumptions are proposed: the location of gas release source is at (0,0,H); wind direction is the forward direction of *x*-axis; wind speed is v m/s; release rate of the source is q kg/s; and atmospheric condition is stable. Under these assumptions, the Gaussian plume model is expressed as follows:

(2) $$c(x,y,z,t)=\frac{D(t)q}{2\pi v\sigma_{y}\sigma_{z}}\exp\left(-\frac{y^{2}}{2\sigma_{y}{}^{2}}\right)\left[\exp\left(-\frac{{\left(z-H\right)}^{2}}{2\sigma_{z}{}^{2}}\right)+\exp\left(-\frac{{\left(z+H\right)}^{2}}{2\sigma_{z}{}^{2}}\right)\right],$$

where c(x,y,z) represents the concentration at location (x,y,z). D(t) is the decay factor containing radioactive decay and deposition. It is an exponential function of time t. When the wind direction is θ, set the release source as the rotation center and then clockwise rotate the concentration distribution c(x,y,z) with degree $\left(90^{{}^{\circ}}-\theta \right)$. If the release source is not at (0,0,H) but $\left(x_{s},y_{s},H\right)$, the concentration distribution c(x,y,z) needs translation transformation.

In the Gaussian plume model, the air contaminant concentration in axis y and z is considered to follow the Gaussian distribution. Therefore, the key parameters of the model are $\sigma_{y}$ and $\sigma_{z}$, which represent the standard deviations that describe the crosswind and vertical mixing of air contaminants and satisfy following relation [26,27]:

(3) $$\left\{ \begin{array}{l} \sigma_{y}=a_{1}\cdot x^{b_{1}}\\ \sigma_{z}=a_{2}\cdot x^{b_{2}} \end{array}\right.,$$

where x means the downwind distance, and $a_{1}$, $a_{2}$, $b_{1}$, and $b_{2}$ depend on the diffusion environment (i.e., it refers to diffusion terrain and other parameters, like atmospheric stability, cloud cover, sunlight and wind). In Gaussian plume model, source term (i.e., release rate and source location) and meteorological data (wind speed and wind direction) can be easily measured. But diffusion coefficients are quite difficult to estimate because they are influenced by various environmental factors. Therefore, the key of data-driven modeling is to update diffusion coefficients. The selection of the state parameters is also the key to the construction of the state transition model. In this paper, system state is expressed by the formula $X_{k}=\{a_{1},b_{1},a_{2},b_{2}\}$.

When applying a particle filter in atmospheric dispersion modeling, it is assumed that F(X)=X, i.e., $X_{k+1}=X_{k}+n_{k}$. Moreover, H(X) can be computed according to Equation (2). After using particle filter and observation to update model parameters, the probability distribution of concentration at each step can be therefore obtained.

In terms of the Gaussian puff model, it is utilized for atmospheric dispersion modeling wherein source releases are instantaneous like an explosion. The advantage of the Gaussian puff model is that it is able to support time-variant wind fields though it cannot model the continuous release. Assuming the release source is located at $\left(x_{s},y_{s},H\right)$ and the center of puff has moved to $\left(x_{c},y_{c},y_{c}\right)$ at time t in wind field W, the equation of Gaussian puff model is shown as follows:

(4) $$c(x,y,z,t)=\frac{D(t)q}{{\left(2\pi \right)}^{3/2}\sigma_{x}\sigma_{y}\sigma_{z}}e^{-\frac{{\left(x-x_{c}\right)}^{2}}{2\sigma_{x}^{2}}}e^{-\frac{{\left(y-y_{c}\right)}^{2}}{2\sigma_{y}^{2}}}\left[e^{-\frac{{\left(z-z_{c}\right)}^{2}}{2\sigma_{z}^{2}}}+e^{-\frac{{\left(z+z_{c}\right)}^{2}}{2\sigma_{z}^{2}}}\right],$$

where q is the instantaneous release quantity and c(x,y,z,t) represents the concentration at (x,y,z) and at time t. D(t) is the radioactive decay and deposition factor. $\sigma_{x}$, $\sigma_{y}$ and $\sigma_{z}$ are diffusion coefficients at *x*-axis, *y*-axis and *z*-axis respectively. They can be computed by the following equations:

(5) $$\left\{ \begin{array}{l} \sigma_{x}=\sigma_{y}=a_{1}\cdot d^{b_{1}}\\ \sigma_{z}=a_{2}\cdot d^{b_{2}} \end{array}\right.,$$

In the Gaussian puff model, diffusion coefficients are the function of total distance d that the puff center has moved. The expression of d is:

(6) $$d=\int_{0}^{t}v(\tau )d\tau ,$$

where v(τ) is the wind speed at time τ. The combination of diffusion coefficients is also utilized as system state in Gaussian puff model $X_{k}=\{a_{1},b_{1},a_{2},b_{2}\}$. State function F(X) also satisfies F(X)=X, while the observation function H(X) can be computed by multi-puff merging.

A single puff cannot calculate the function H(X) because a Gaussian puff model cannot simulate the dispersion process of a continuous release. Fortunately, the gas plume can be regarded as the combination of gas puffs that are released from a source with an infinitely little temporal interval, which is named multi-puff merging. Therefore, a series of puffs can be merged to approximately substitute a plume. When the release rate q is time-variant, q(t) can be discretized by substituting the plume by a series of puffs with different release quantities. Let $p=\{x_{s},y_{s},z_{s},q,t_{start}\}$ represent a single puff containing all essential parameters for atmospheric dispersion modeling, where p can be described as a puff released from $\left(x_{s},y_{s},z_{s}\right)$ at time $t_{start}$ with instantaneous release quantity q. The corresponding function $c_{p}(x,y,z,t,p)$ represents the concentration of location (x,y,z) at time t of puff p. Thus, the whole dispersion process can be expressed as:

(7) $$c(x,y,z,t)\approx \sum_{i=1}^{n}c_{p}(x,y,z,t,p_{i}),$$

where $p_{i}$ means the ith puff divided from the plume. By substituting the plume by puffs, it is feasible to model the atmospheric dispersion whose release rate is varying or whose wind field is dynamic. Thus, the observation function H(X) can be easily derived via this approach.

The structure and workflow of the particle filter as well as the detailed procedure of data assimilation are included in an independent section of our previous studies. For simplicity, these contents will not be introduced again and interested readers are referred to those references [3,28].

### 2.3. Error Propagation Detection

In numerical computing, error propagation would lead to devastating results. For example, a Patriot missile failed to intercept its target, and this caused many casualties during the Gulf War because of a floating-point computational error. In this paper, errors of atmospheric dispersion modeling and simulation are classified into two categories: measurement error ε and floating-point error ϵ. Measurement errors usually represent the errors generated by monitoring devices. They usually include errors in source term information (release rate and WGS84 coordinate), meteorological data (wind speed and wind direction), concentration data, etc. Nevertheless, floating-point errors are generated during the numerical computation due to the limits of memory and the expression of floating-point numbers. Since most real numbers cannot be expressed precisely in a computer, floating-point errors may appear in any places of the atmospheric dispersion simulation, like wind field generation, dispersion equation calculation and particle filtering iteration. In this paper, an error propagation detection method based on the relative error exponent is developed. The exponent of relative error e can be calculated by $r=\left\lfloor \log_{2}e\right\rfloor$. Consequently, this method can find the potential floating-point instability problems during the computation, which can assist environmental protection authorities to evaluate whether the results are of high-confidence.

In this paper, the floating-point errors and measurement errors are combined in the source term parameters, wind field and concentration data. To fit the concentration measurements, the integration of particle filtering and error propagation detection is then used to update the system state of the atmospheric dispersion process. The estimated concentration distribution and error analysis are finally computed.

Errors are highly possible to seriously affect the accuracy of results and they are inevitable because of the limitations of sensors and computers. Therefore, any atmospheric dispersion modeling results without a high-confidence error analysis are not convincing. The proposed error detection method is able to verify that to what extent the results are seriously influenced by errors. In order to explain the error propagation detection, several definitions should be introduced firstly [24]. For a variable, x means “computational value”, which is the value stored in the computer memory. Computational value is limited by measurement error and floating-point error. “Actual value” $\hat{x}$ means the value of the variable in reality with no error (infinite precision). Absolute error ${\hat{\Delta }}_{x}=\hat{x}-x$ represents the difference between actual value and computational value with infinite precision, which can also be expressed as ${\hat{\Delta }}_{x}=\varepsilon_{x}+\epsilon_{x}$ (i.e., sum of measurement error and floating-point error). Relative error $\Delta_{x}$ is computed as $\Delta_{x}=|{\hat{\Delta }}_{x}/x|$. Obviously, when computational value is close to zero, it is possible to generate high relative error. For example, consider the addition operation:

(8) $$\hat{x}+\hat{y}=x+{\hat{\Delta }}_{x}+y+{\hat{\Delta }}_{y}=(x+y)+({\hat{\Delta }}_{x}+{\hat{\Delta }}_{y}),$$

The computational result of this expression is x+y, and the absolute error is ${\hat{\Delta }}_{x}+{\hat{\Delta }}_{y}$. Therefore, the relative error can be computed by equation $\left|\left({\hat{\Delta }}_{x}+{\hat{\Delta }}_{y})/(x+y\right)\right|$. When x=10,000, ${\hat{\Delta }}_{x}=1$, y=−9999.9 and ${\hat{\Delta }}_{y}=1$, both $\Delta_{x}$ and $\Delta_{y}$ are relatively little (close to $10^{-4}$). However, the relative error of the sum $\Delta_{x+y}$ reaches 20, which means the relative error is $10^{5}$ times greater than the original ones.

In atmospheric dispersion modeling and simulation, errors usually contain measurement errors and floating-point errors. Measurement errors exist in all the sampled data from monitoring devices. Therefore, all the input measurements have initial errors. In terms of floating-point errors, most real numbers cannot be expressed precisely in a computer because they are limited by the floating-point format. When a program runs, the floating-point errors will propagate gradually. As concluded in the example, addition operations may cause huge relative error. Furthermore, errors could also be propagated because of multiplication, division, exponential, operations, etc. As a result, a detailed list of error propagation rules for relative error inspection is important in atmospheric dispersion modeling and simulation.

### 2.4. Relative Error Exponent

For one thing, the computer needs huge resources to record the actual values with no error. For another thing, some irrational numbers are impossible to be recorded in memory. Thus, the precise error of each variable cannot be calculated in a normal program. To address this problem, a method of estimating the exponent of relative error r is proposed to classify the error of each variable. Firstly, according to the property of monitoring device and its floating-point value, the exponent $e_{x}$ of variable x (calculated by equation $\left\lfloor \log_{2}x\right\rfloor$) and its relative error exponent $r_{x}$ (calculated by equation $\left\lfloor \log_{2}\Delta \right\rfloor$) are determined. Moreover, exponent of relative measurement error $r_{x}^{\varepsilon }$ can be easily calculated, and initial exponent of relative floating-point error $r_{x}^{\epsilon }$ can also be estimated precisely. However, it may take too much time for error estimation. Thus, it is assumed that the initial exponent of relative floating-point error $r_{x}^{\epsilon }$ of a “float” variable is −20, and that of a “double” variable is −50. The initial exponent of relative error is the maximum of $r_{x}^{\epsilon }$ and $r_{x}^{\varepsilon }$. Furthermore, error propagation rules of operations appearing in data-driven atmospheric dispersion modeling (addition, multiplication, inversion, exponential, square root, and sine) are shown in Table 1. Addition and multiplication can be found anywhere during computation, while inversion, exponential and square root are used in Gaussian models and radioactive decay factors. Sine functions are essential in wind field generation.

In terms of addition, the relative error of addition has also been analyzed before. Consequently, the relative error $\Delta_{x+y}$ can be obtained by following equation:

(9) $$\Delta_{x+y}=\left|\frac{{\hat{\Delta }}_{x}+{\hat{\Delta }}_{y}}{x+y}\right|\leq \frac{\left|{\hat{\Delta }}_{x}\right|+\left|{\hat{\Delta }}_{y}\right|}{\left|x+y\right|}=\frac{\left|x\Delta_{x}\right|+\left|y\Delta_{y}\right|}{\left|x+y\right|},$$

As shown in this equation, the value of relative error exponent $r_{x+y}$ depends on the values of $\left|x\Delta_{x}\right|$ and $\left|y\Delta_{y}\right|$. Thus, the exponent can be estimated by $r_{x+y}=\max(e_{x}+r_{x},e_{y}+r_{y})-e_{x+y}$. As for multiplication, the derivation process is similar to addition. Relative error $\Delta_{xy}$ can be calculated first:

(10) $$\Delta_{xy}=\left|\frac{(x+{\hat{\Delta }}_{x})(y+{\hat{\Delta }}_{y})-xy}{xy}\right|=\left|\frac{y{\hat{\Delta }}_{x}+x{\hat{\Delta }}_{y}+{\hat{\Delta }}_{x}{\hat{\Delta }}_{y}}{xy}\right|\leq \frac{\left|y{\hat{\Delta }}_{x}\right|+\left|x{\hat{\Delta }}_{y}\right|+\left|{\hat{\Delta }}_{x}{\hat{\Delta }}_{y}\right|}{\left|xy\right|},$$

Because $\left|{\hat{\Delta }}_{x}\right|=x\Delta_{x}$ and $\left|{\hat{\Delta }}_{y}\right|=y\Delta_{y}$, we have:

(11) $$\Delta_{xy}\leq \frac{\left|yx\Delta_{x}\right|+\left|xy\Delta_{y}\right|+\left|x\Delta_{x}y\Delta_{y}\right|}{\left|xy\right|}=\left|\Delta_{x}\right|+\left|\Delta_{y}\right|+\left|\Delta_{x}\Delta_{y}\right|,$$

Thus, the relative error exponent of multiplication is $r_{xy}=\max(r_{x},r_{y},r_{x}+r_{y})$. Division can be decomposed into multiplication and inversion operations. In terms of the inversion 1/x, the derivation equation of $\Delta_{1/x}$ is:

(12) $$\Delta_{1/x}=\left|\frac{dx^{-1}}{dx}\frac{{\hat{\Delta }}_{x}}{x^{-1}}\right|=\left|\frac{1}{x^{2}}\frac{x\Delta_{x}}{x^{-1}}\right|=\left|\Delta_{x}\right|,$$

Therefore, the relative error exponent of inversion operation is $r_{1/x}=r_{x}$. The calculation of exponential operation needs approximation. The relative error $\Delta_{\exp(x)}$ can be expressed by the following equation:

(13) $$\Delta_{\exp(x)}=\left|\frac{{\hat{\Delta }}_{\exp(x)}}{\exp(x)}\right|\approx \left|\frac{d\exp(x)}{dx}\frac{{\hat{\Delta }}_{x}}{\exp(x)}\right|=\left|x\Delta_{x}\right|,$$

Then, the expression of relative error exponent $r_{\exp(x)}$ of exponential operation $r_{\exp(x)}=e_{x}+r_{x}$ can be obtained. In terms of square root operation, its relative error $\Delta_{\sqrt{x}}$ can be calculated by:

(14) $$\Delta_{\sqrt{x}}=\left|\frac{{\hat{\Delta }}_{\sqrt{x}}}{\sqrt{x}}\right|\approx \left|\frac{d\sqrt{x}}{dx}\frac{{\hat{\Delta }}_{x}}{\sqrt{x}}\right|=\left|\frac{1}{2\sqrt{x}}\frac{x\Delta_{x}}{\sqrt{x}}\right|=\left|\frac{\Delta_{x}}{2}\right|,$$

so the exponent of relative error is $r_{\sqrt{x}}=r_{x}-1$. In order to estimate the relative error exponent of sine function, the relative error also needs some transformation so that it can be expressed by x and $\Delta_{x}$. The derivation equation is:

(15) $$\Delta_{\sin(x)}=\left|\frac{{\hat{\Delta }}_{\sin(x)}}{\sin(x)}\right|\approx \left|\frac{d\sin(x)}{dx}\frac{{\hat{\Delta }}_{x}}{\sin(x)}\right|=\left|\frac{\cos(x)x\Delta_{x}}{\sin(x)}\right|,$$

Therefore, the relative error exponent of sine function is $r_{\sin(x)}=e_{\cos(x)x/\sin(x)}+r_{x}$. These rules cover all operations used in this study. They are written inside the program to trace the error propagation and give the final analysis. In summary, the error propagation rules of all necessary operations are shown in Table 1.

## 3. Application: Indianapolis Field Study

### 3.1. Scenario Introduction

**Figure 1:**
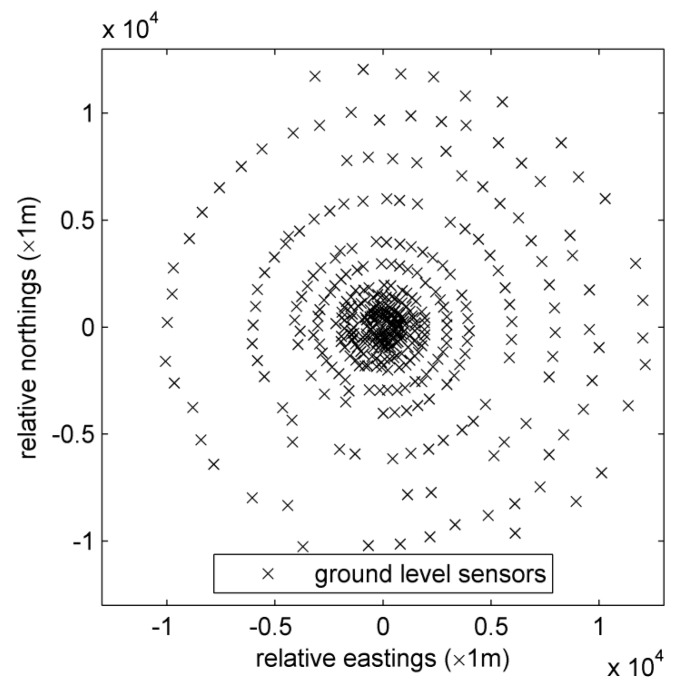
Distribution of ground-level concentration monitoring sensors.

The Indianapolis experiment was implemented from 16 September to 12 October in 1985 [29]. Researchers used sulfur hexafluoride (SF_6_) to trace the dispersion plume emitted from an 83.8-m height stack. SF_6_ is a kind of artificial inert gas that is colorless, odorless, non-flammable, and non-toxic. Furthermore, the concentration of SF_6_ is negligible in the atmosphere because it is only synthesized in the laboratory. Therefore, background concentrations can be ignored in the environment, which is similar to the hazardous gas accidently leaked from the chemical industry park or nuclear power plant. These features make SF_6_ a perfect gas for dispersion tracing, and it is a safe substitute of hazardous gas trace experiments. SF_6_ was released from the stack built at the Perry K power plant in Indianapolis, Indiana, United States. The WGS84 coordinate of the release source is (39.8 E latitude, 86.2 E longitude), and its UTM coordinates are (N4401.59 km, E571.40 km). The elevation of this power plant is 214 m. From 16 September to 12 October, monitoring stations collected sampled data for about 170 h. Eight or nine hours of SF_6_ concentration data were available on each day. Meteorological data were sampled from a 94 m height monitoring tower in a bank and three other 10 m height monitoring towers at urban, suburban, and rural locations, respectively. About 160 ground-level concentration monitoring stations were established in arcs at distances ranging from 0.25 to 12.0 km from the release source. Observation data include the concentration measurements, meteorological observations, locations of all meteorological monitoring towers and ground-level monitoring sensors. Distribution of all sensors can be viewed in Figure 1.

The Indianapolis case is one of the most complete gas trace experiments available since the measurements of this experiment are sufficient and SF_6_ is an appropriate low-risk hazardous gas substitute. Therefore, this experiment was applied in our study to test the performance of the proposed method. The sampled SF_6_ concentration datasets are used for data-driven modeling. Because the measurement errors of Indianapolis experiment were not given, some assumptions concerning measurement error are made and then the performance of error propagation is analyzed.

### 3.2. Parameters Configuration

The experiment implemented in this paper uses the sampled data of the Indianapolis field experiment collected on 20 September in 1985 for data-driven modeling and error detection analysis. Four test cases are set to test the performance of the Gaussian plume model, Gaussian multi-puff model, and the corresponding two PF models. Concentration data from 10:00 to 18:00 were available. Wind speed and wind direction were sampled each hour, and the wind field was quite stable that day. Further, the release rate of the stack in Perry K power plant remained constant at 4.65 g/s. These conditions imply that the Gaussian plume model is also quite suitable in this case.

**Figure 2:**
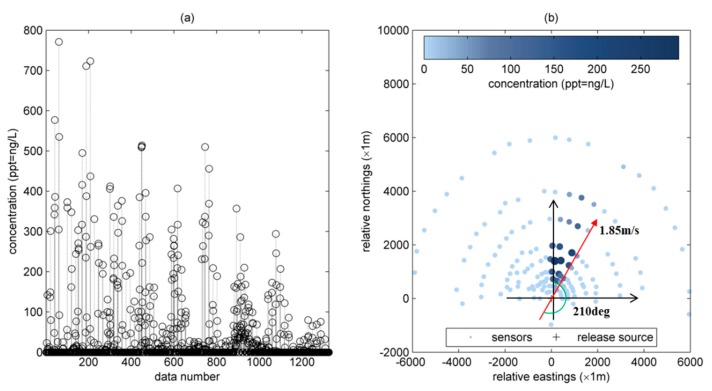
SF6 concentration data at 20 September. (**a**) 1326 concentration records; (**b**) distribution of monitoring stations and concentration distribution at 10:00).

A total of 1326 records were sampled by these monitoring sensors in this day (see Figure 2a). The label of *x*-axis ‘data number’ in Figure 2a means the measurement sequence in the 1326 records. The distribution of monitoring sensors that were operating on 20 September in 1985 is shown in Figure 2b. In this figure, it can be seen that the available ground-level sensors are arranged as an arc to the north of the release source (83.8 m stack at the Perry K power plant). Concentration data are sampled each hour during this day. Figure 2b also demonstrates the concentration distribution at 10:00, where the size and darkness of the point represent the value of concentration. The bigger point size represents higher concentration value at the corresponding monitoring sensor. Similarly, the darker points also represent a higher concentration value. Obviously, the concentration values sampled from the northeast direction to the release source are relatively high, which has an acceptable agreement with the measured wind direction (i.e., the averaged wind direction is 210° and the averaged wind speed is 1.85 m/s).

Gaussian plume model, Gaussian multi-puff model, Gaussian-PF plume model and Gaussian-PF multi-puff model are used in experiments to reconstruct the process of SF_6_ atmospheric dispersion. While Gaussian plume and puff models are traditional approaches whose diffusion coefficients are constant, Gaussian-PF plume and Gaussian-PF multi-puff models are data-driven modeling approaches. Error propagation detection is applied to analyze whether the results are seriously influenced by errors. The diffusion coefficients of two traditional approaches are determined according to the atmospheric stability class, to recover the whole dispersion process. In terms of Gaussian-PF models, the diffusion coefficients keep changing in order to fit the observation data. The ranges of diffusion coefficients in Gaussian-PF models are listed in Table 2.

As for initial errors, according to the experimental description of the Indianapolis case, the resolution of concentration monitoring stations is 1 ppt. However, the measurement error is not given in the corresponding reports. Thus, it is assumed that the monitoring devices have quite high accuracies. Consequently, the error of concentration monitoring stations is assumed to be 1 ppt (equal to the sensor resolution). Similarly, the measurement error of wind direction is assumed to be 1 degree and the error of wind speed is 0.01 m/s. Notice that the wind direction is a relative value so that the relative error of wind direction d is a constant value 1/360 but not 1/d (exponent of its relative error is −9).

## 4. Results and Discussion

The concentration generated in the same monitoring station declines with time (see Figure 2a). This seems unreasonable because the meteorological measurements show that the wind direction only showed a little fluctuation during this period and the wind speed only increased slightly. Moreover, the release rate measured by the monitoring sensor installed on the stack also shows that it remained at the same level. Therefore, the reason that causes the decrease of concentration is the environmental conditions, which is affected largely by atmospheric stability, cloud cover, sunlight and other complex factors [27]. The factors of atmospheric stability, cloud cover, sunlight would generate an effect of plume rise [30]. It is worth noting that the plume rise is beneficial to reduce the concentration of pollutants on the ground. Moreover, atmospheric stability, sunlight and terrain greatly influence the determination of dispersion coefficients [31,32,33]. Dispersion coefficients are of vital importance to dispersion simulation. The disadvantage of traditional plume models is that diffusion coefficients cannot be directly calculated.

**Figure 3:**
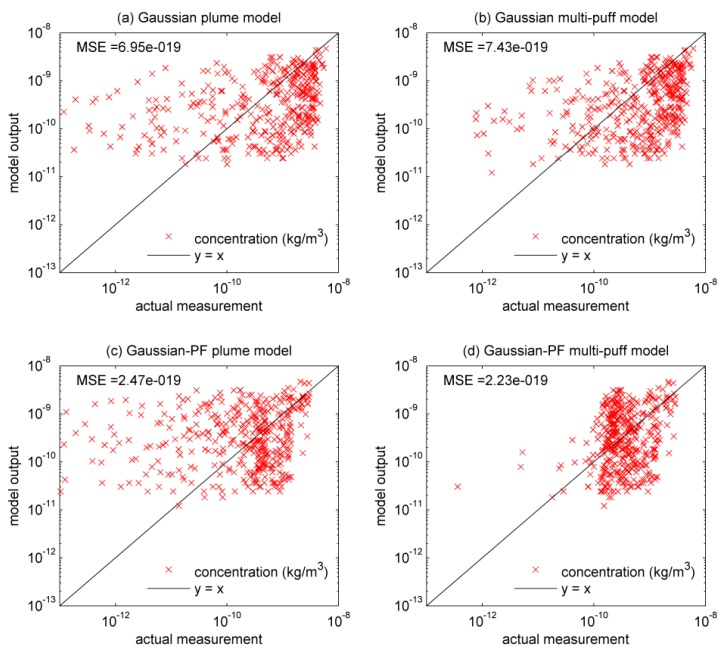
Comparisons of real measurements and modeled concentrations calculated by (**a**) Gaussian plume model; (**b**) Gaussian multi-puff model; (**c**) Gaussian-PF plume model, and (**d**) Gaussian-PF multi-puff model.

**Figure 4:**
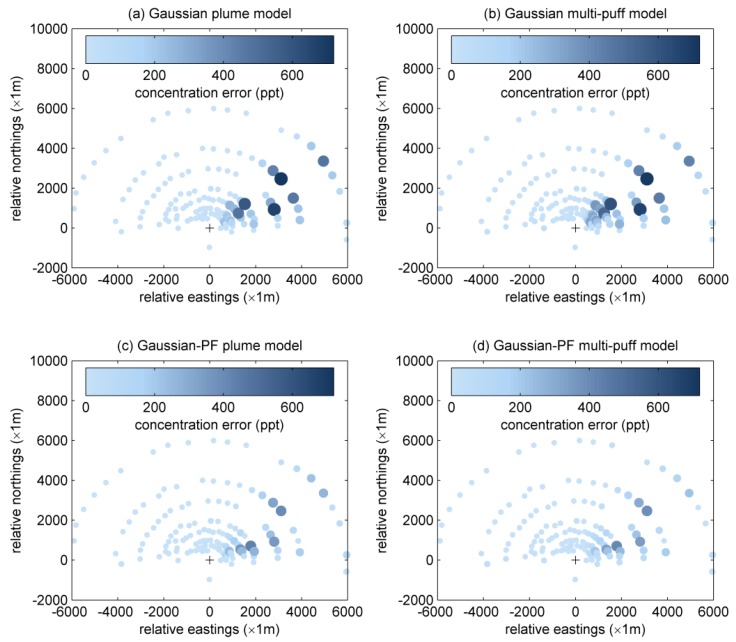
Distribution of concentration error of monitoring sensors at 10:00 calculated by (**a**) Gaussian plume model; (**b**) Gaussian multi-puff model; (**c**) Gaussian-PF plume model, and (**d**) Gaussian-PF multi-puff model.

The results of using the traditional Gaussian plume model without data-driven modeling to recover the SF_6_ atmospheric dispersion are shown in Figure 3a and Table 3. The mean squared error (MSE) of the traditional Gaussian plume model is $6.95\times 10^{-19}$. Meanwhile, the correlation coefficient (*r*) between observations and model predictions is 0.5409. The mean value of real measurements, mean value of model predictions, standard deviation value of real measurements and standard deviation value of model predictions are $2.0139\times 10^{-10}$, $4.3313\times 10^{-10}$, $5.3745\times 10^{-10}$ and $9.6761\times 10^{-10}$ respectively. Figure 4a illustrates the concentration error (absolute difference between measured concentration and modeled concentration) distribution at 10:00. Obviously, the Gaussian plume model shows quite high error at some sensors.

Figure 3b and Table 3 display the results of the traditional Gaussian multi-puff model without particle filtering. In this figure, the modeling results of traditional Gaussian plume and puff models are quite similar, which means merging a series of puffs to substitute a plume is a feasible approach. However, both traditional Gaussian plume and multi-puff models cannot change the diffusion coefficients during the simulation process. The MSE of Gaussian multi-puff model is $7.43\times 10^{-19}$, even higher than plume model. Meanwhile, the correlation coefficient (*r*) between observations and model predictions is 0.6046, which outperforms the value of Gaussian plume model. The mean value of real measurements, mean value of model predictions, standard deviation value of real measurements and standard deviation value of model predictions are $2.0139\times 10^{-10}$, $5.0905\times 10^{-10}$, $5.3745\times 10^{-10}$ and $1.0072\times 10^{-9}$ respectively. Seen from Figure 4b, traditional Gaussian puff model shows high error at some sensors, which is similar to the traditional Gaussian plume model.

In terms of PF-based methods, Figure 3c and Table 3 illustrate the results of Gaussian-PF plume. Obviously, data-driven modeling based on particle filtering has played an important role during computing. The accuracy of modeled concentrations improves markedly. The MSE of the final result is $2.47\times 10^{-19}$, which is significantly less than the MSEs of traditional Gaussian models. Meanwhile, the correlation coefficient (*r*) between observations and model predictions is 0.4577, which experiences an obvious decrease compared to that of traditional Gaussian plume model. The mean value of real measurements, mean value of model predictions, standard deviation value of real measurements and standard deviation value of model predictions are $2.0139\times 10^{-10}$, $2.1963\times 10^{-10}$, $5.3745\times 10^{-10}$ and $4.1629\times 10^{-10}$, respectively. Figure 4c shows that the concentration error at 10:00, which has been reduced significantly comparing to traditional models.

As shown in Figure 3d and Table 3, the results of Gaussian-PF multi-puff model is also satisfactory. The MSE of this model is only $2.23\times 10^{-19}$. Therefore, Gaussian-PF multi-puff model has the optimal fit to the actual measurements. Meanwhile, the correlation coefficient (*r*) between observations and model predictions is 0.5211, which also experiences an obvious decrease compared to that of traditional Gaussian multi-puff model. The mean value of real measurements, mean value of model predictions, standard deviation value of real measurements and standard deviation value of model predictions are $2.0139\times 10^{-10}$, $2.5882\times 10^{-10}$, $5.3745\times 10^{-10}$ and $3.7687\times 10^{-10}$ respectively. However, as shown in Table 4, the Gaussian-PF multi-puff model needs quite a long execution time. Therefore, when meteorological conditions are relatively stable, Gaussian-PF plume model is appropriate since it performs well in both fitness and execution time. However, if wind direction and wind speed are unstable and dynamic, Gaussian-PF multi-puff model is the only choice that is able to fit measurements well [34].

As seen in Figure 3, Table 3 and Table 4, the Gaussian-PF models perform better in MSE while the traditional Gaussian-based models have better correlation coefficients. The measured concentration values obey the approximate Gaussian distribution and the concentration curve computed by Gaussian-based models also conforms to the Gaussian distribution. Thus, the correlation coefficient between real measurements and predictions of Gaussian-based models is higher. However, the predicted error is larger because Gaussian-based models are simple. For the Gaussian-PF models, in order to approximate the real concentration value, the Gaussian-PF models have to change the original Gaussian distribution curve. So the correlation coefficient between real measurements and predictions of Gaussian-PF models decreases. The purpose of the proposed method is to reduce the prediction error, and therefore the proposed method is acceptable. As for computational time, the computational efficiency is sacrificed for the improvement of prediction accuracy. The addition of the particle filter and error propagation detection makes the proposed models very computational expensive. However, it is noteworthy that the unit of computational time is ms and most of the computational-time results (e.g., 1388.19, 2453.13 and 120,040.87 ms) are acceptable. Moreover, when applied into practical utilization, it is better for users to consider the practical needs and purposes. For example, if users require accurate prediction results in a limited time, Gaussian-PF plume model would be the optimal choice. However, if the wind field is varying and the users do not care about the calculation time, Gaussian-PF multi-puff model would be the better choice.

**Figure 5:**
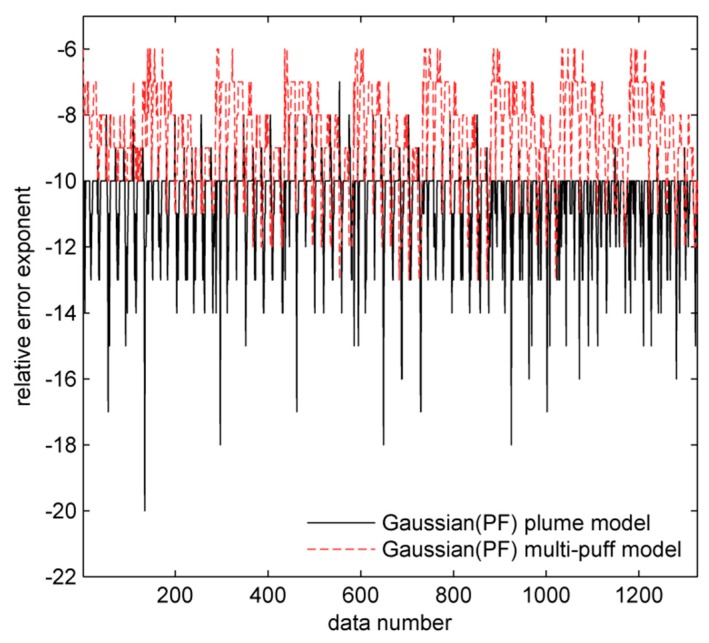
Relative error exponents of concentrations.

The results of error analysis are shown in Figure 5. After experiments, it is found that the final relative error exponents of traditional models and their corresponding data-driven modeling methods are same because randomly generating system states can eliminate the historical error at each step. Therefore, data-driven modeling method based on particle filtering will not accumulate errors. In this figure, it is noteworthy that values of relative error exponent are all integers. This is because the definition of relative error exponent $r_{x}$ (calculated by equation $\left\lfloor \log_{2}\Delta \right\rfloor$ and it is an integer) and its operations (i.e., operations of plus and minus in third line of Table 1). Furthermore, both plume and multi-puff model do not have large relative errors after computing. After comparing the final relative error exponents of plume model and multi-puff model, it is obvious that Gaussian plume model and its corresponding data-driven modeling method have less probability to cause floating-point unstable problem.

**Figure 6:**
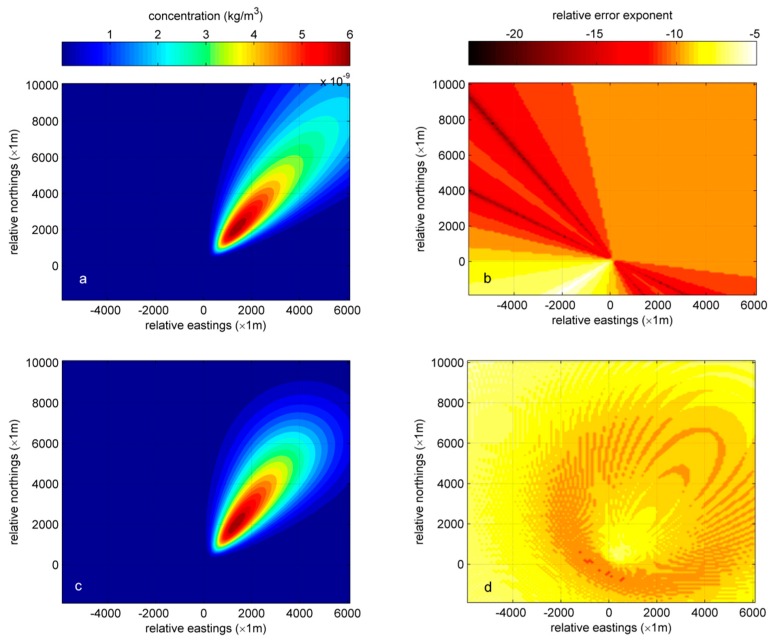
Relative analysis results of Gaussian plume model, Gaussian multi-puff model and their PF variation. (**a**) Modeled concentration distribution at 11:00; (**b**) Relative error exponent distribution matches to modeled concentration distribution; (**c**) Modeled concentration distribution at 11:00; (**d**) Relative error exponent distribution matches to modeled concentration distribution).

Figure 6a shows the distribution of concentration at 11:00 using Gaussian-PF plume model. Figure 6b illustrates the corresponding relative error exponents of calculated concentrations. Because the expression of Gaussian plume model is quite simple, the relative error exponent mostly depends on the wind direction. As shown in this figure, the relative error exponents of calculated concentrations at crosswind direction are low, while the relative error exponents at upwind direction are quite high. The concentration at upwind direction is usually very close to zero, so it is reasonable that its corresponding error exponent is high. Therefore, Gaussian plume model is stable because the maximum relative error exponent is only −5 and it will not increase with time.

The error analysis results of Gaussian-PF multi-puff model and its corresponding data-driven modeling method are also shown in Figure 6. Figure 6c demonstrates the concentration distribution of Gaussian-PF multi-puff model at 11:00 and its relative error exponent distribution is shown in Figure 6d. A significant difference between Gaussian plume and Gaussian multi-puff model is that the error propagates with time in Gaussian multi-puff model while it does not increase in the Gaussian plume model. Thus, in the Gaussian multi-puff model, it is obvious that points far from release source generally have high relative errors because puffs need more time to reach these points. However, points that are very close to the release point also have high relative errors because their downwind distances are too short (very close to zero) to avoid errors.

As can be seen in Table 4, after using error detection, the computational times of the Gaussian multi-puff and Gaussian-PF multi-puff models become very long, while error detection has relatively little influence on Gaussian plume model. The computational time of Gaussian-PF plume model is shorter than the Gaussian multi-puff model. Thus, Gaussian-PF plume model is an appropriate choice when the wind field is stable. The Gaussian-PF multi-puff model has the highest accuracy, quite low error and long computational time, so it might be a better option when the time requirement is not so strict. More importantly, the utilization of Equation (3) for the diffusion coefficient is usually applied in the case of the open terrain, while the Indianapolis experiment was performed in urban terrain [35]. Actually, the focus of this paper is to improve the prediction performance of atmospheric dispersion simulation. As seen from experimental results, it is concluded that the data-driven method based on particle filter can improve the prediction ability of Gaussian-based models and error propagation detection can inform users whether or not the prediction results are of high confidence. Thus, the results imply that our proposed method is feasible in actual experiments. For further improving the fitting results in Figure 3 and Table 3, the diffusion coefficients applied in urban terrain should be used. Interested readers can refer to our future work.

## 5. Conclusions

In this paper, a data-driven method using a particle filter is developed to improve the accuracy of air contaminant dispersion predictions based on Gaussian-based models. In order to judge whether the predicted results are convincing, an error propagation detection method is also proposed in this paper to analyze the influence of measured or computational errors. In terms of data-driven modeling, diffusion coefficients are regarded as the system states, and particle filtering is then used to update diffusion coefficients during each-step calculation. As for error propagation detection, six rules are listed to inspect the error propagation of essential operations during numerical computing. Then, a series of experiments are designed based on Indianapolis Field Study to test the performances of the proposed data-driven modeling method. Compared to traditional Gaussian-based models (the MSEs of Gaussian plume model and Gaussian multi-puff model are $6.95\times 10^{-19}$ and $7.43\times 10^{-19}$ respectively), the proposed methods based on PF and error propagation detection witness a significant improvement in prediction accuracy and provide convincing results (the MSEs of Gaussian-PF plume model and Gaussian-PF multi-puff model are $2.47\times 10^{-19}$ and $2.23\times 10^{-19}$ respectively) though requiring more computational time (1.39 s for Gaussian-PF plume model; 2.45 s for Gaussian-PF plume model with error detection; 120.04 s for Gaussian-PF multi-puff model; 3672.95 s for Gaussian-PF multi-puff model with error detection). In conclusion, data-driven modeling methods have higher accuracy but longer computational time. However, when error detection is applied in the program, the Gaussian-PF plume is still faster than the traditional puff model, while the computational time of Gaussian-PF multi-puff model becomes extremely long. The results reveal that when applied into practical utilization, it is better for users to consider the practical needs and purposes. If users require accurate prediction results in a limited time, Gaussian-PF plume model would be the optimal choice. However, if the wind field is varying and the users do not care about the calculation time, Gaussian-PF multi-puff model would be the better choice. Therefore, the proposed methods provide strong support for the prediction of air contaminant dispersion and emergency management in chemical clusters.

Future works should include implementing the field experiment in a chemical cluster to verify the data-driven method in real situations, improving the computational efficiency of the proposed methods and dynamic modeling of the wind field for a more accurate prediction of the atmospheric dispersion model.

## Figures and Tables

**Table 1:** Error propagation rules of each operation.

Operation	Expression	Exponent of Relative Error
Addition	x+y	$r_{x+y}=\max(e_{x}+r_{x},e_{y}+r_{y})-e_{x+y}$
Multiplication	xy	$r_{xy}=\max(r_{x},r_{y},r_{x}+r_{y})$
Inversion	1/x	$r_{1/x}=r_{x}$
Exponential	exp(x)	$r_{\exp(x)}=e_{x}+r_{x}$
Square root	$\sqrt{x}$	$r_{\sqrt{x}}=r_{x}-1$
Sine	sin(x)	$r_{\sin(x)}=e_{\cos(x)x/\sin(x)}+r_{x}$

**Table 2:** Ranges of diffusion coefficients in Gaussian-PF models.

Diffusion Coefficient	Range (Gaussian-PF Multi-Puff and Gaussian-PF Plume)
$a_{1}$	(0.1,0.5)
$b_{1}$	(0.8,1.0)
$a_{2}$	(0.9,1.1)
$b_{2}$	(0.4,0.6)

**Table 3:** Correlation coefficients, the mean values and standard deviations corresponding to Figure 3. (*r* means correlation coefficients between observations and model predictions; Mean_real and Mean_model represent mean value for real observations and model respectively; similarly, Std_real and Std_model represent standard deviation for real observations and model.).

	*r*	Mean_Real	Mean_Model	Std_Real	Std_Model
(a)	0.5409	$2.0139\times 10^{-10}$	$4.3313\times 10^{-10}$	$5.3745\times 10^{-10}$	$9.6761\times 10^{-10}$
(b)	0.6046	$2.0139\times 10^{-10}$	$5.0905\times 10^{-10}$	$5.3745\times 10^{-10}$	$1.0072\times 10^{-9}$
(c)	0.4577	$2.0139\times 10^{-10}$	$2.1963\times 10^{-10}$	$5.3745\times 10^{-10}$	$4.1629\times 10^{-10}$
(d)	0.5211	$2.0139\times 10^{-10}$	$2.5882\times 10^{-10}$	$5.3745\times 10^{-10}$	$3.7687\times 10^{-10}$

**Table 4:** Average computational time of each model.

Model Name	Computational Time (ms)	
	Native	With Error Detection
Gaussian plume model	134.67	154.41
Gaussian multi-puff model	285.53	6162.30
Gaussian-PF plume model	1388.19	2453.13
Gaussian-PF multi-puff model	120,040.87	3,672,952.18
